# On the General Use of Chloroform

**Published:** 1857-03

**Authors:** 


### SELECTIONS.

*On the General Use of Chloroform.* By Dr. M'Leod. In the Medical and Surgical Society of London (May, 1856,) Dr. M'Leod read the following paper, *On the General Use of Chloroform*:—

The author began by remarking on the necessity which existed for all surgeons clearly making up their minds on this important subject, and thoroughly studying the question in all its bearings. He proposed to run rapidly over the different points of practical moment presented by a consideration of anæsthesia; and to submit the question as clearly a possible before the Society, with the view of eliciting from its members an expression of their opinion on the subject. Dr. M'Leod referred at the outset to the experiments of Dr. Nunnely and Dr. Simpson, instituted for the purpose of determining the relative value of different anæsthetics, and stated his intention of confining his observations, in what followed, to chloroform, as being the only anæsthetic of practical value. He then reviewed the different hypotheses which had been started, to explain the physiological action of anæsthetics when inhaled, and gave his adhesion to that view which ascribed it to absorption into the blood, and its being thereby carried to the nervous centres. The fact that both the chloroform and ether can be detected in the flesh and blood for a considerable period after they have been inhaled, the author thought, went a considerable way to support that view. Dr. M'Leod then dwelt upon the modes in which anæsthetics, when inhaled, might cause death. He showed that in those cases which had ended fatally, as well as in experiments conducted on animals to determine the question, the most constant appearances were these:—1. A highly congested state of the pulmonary tissues. 2. An engorged state of the right side of the heart, and an empty state of the left; in other cases a flaccid condition of the whole organ. 3. A congested state of the brain. These, with an altered condition of the blood itself, seemed to be fairly attributable or referrible to the drug. Death, then, had been ascribed to:—1. Asphyxia, caused, according to some, by the arrest of the chemical changes carried on in the lungs; and, according to others, by the capillary vessels of the lungs. 2. To coma, caused by the action of the vapor in the nervous centres. And, 3. To syncope, caused either through the various centres, or from the local action of the overcharged blood in the heart itself. From a careful consideration of the fatal causes, death, the author thought, was sometimes due to one of these modes, and sometimes to another, and at times to two or more of them combined. He showed that all arose from the employment of the vapor too little diluted with atmospheric air, and were to be avoided by carefully guarding against such an error in the administration. Dr. M'Leod next alluded to the fallacy of allowing theoretical notions, as to what parts of the nervous system are at any particular period of the administration being implicated, or as to how many drops are necessary to produce such and such effects, to interfere in the practical employment of anæsthetics. Such attempts only withdraw the mind from the real points of importance, and lead to erroneous practice. He contended that all apparatus was not only uncalled for, but absolutely injurious, as tending to frighten the patient, and prevent the escape of the expired breath. He said it mattered not whether we measured the amount of the liquid the patient had inhaled or not, so long as we are guided by effects. The propriety of keeping the patient from food for some hours previous to the administration of the anæsthetic, the necessity for quiet during the administration, and of allowing a free circulation of pure air around the patient, were dwelt upon, and great weight was put upon the recumbent position being assumed in all cases during the exhibition. The removal of all constrictions of dress about the neck and chest was insisted on, as well as the necessity of observing the temperature of the apartment, as Dr. Snow had shown how great a difference existed in the amount of vapor set free at different elevations of temperature. The advantage of bringing the patient rapidly under the influence of the drug, while a large amount of atmospheric air was admitted, was pointed out; and the author proceeded to show that the discrepancy of opinion as to the '*upholding effects*' of chloroform, arose from the degree of action established; that, if not carried beyond a certain point, the effect was certainly of a supporting character; and that the depression spoken of by observers was the result of a larger amount of vapor being administered than was justifiable. The respiration was shown to be the great guide in the administration of chloroform. The eye, too, being upturned and fixed, afforded no information as to the establishment of the action, but neither the pulse nor the pupil communicated anything. The propriety of observing the color of the lips and countenance, and also the flow of the blood from the cut vessels, was declared to be of consequence as affording indications of the approach of syncope. The author stated his conviction that age, sex, diathesis, idiosyncrasy, were matters of indifference in administering chloroform, if we were guided by effects. Having referred to the combined use of chloroform and ether, the author went on to speak of the proper steps to be taken in the event of an overdose. As the chief danger was seen to arise from the use of the vapor in a state of too great density, or from its accumulation in the system, the great remedy was shown to be the free admission of pure air, and the employment of artificial respiration, if the patient was too deeply affected to work off the overcharge by his own exertions. The wonderful manner in which the respiratory movements may be excited by galvanism was then referred to. The method of raising the epiglottis by the finger, or by drawing forward the tongue, as recommended by M. Regnault, was strongly advocated, and the assistance obtained in producing the desired result by dashing cold water on the face and chest noticed. If the danger arose from syncope, the propriety of applying stimulants to the nostrils, and using them by the rectum, or the direct stimulation of the heart by needles, or the actual cautery, were pointed out. The method of inverting the patient, recommended by M. Nelaton in such cases, was also detailed. No fluids should be given by the mouth for some time, till the patient had become conscious.

The author summed up this part of the subject by recommending in all cases the admission of a stream of fresh air, the drawing forward of the tongue, and the application of cold water to the face and chest. If death appeared to approach by syncope, stimulate the heart in one of the ways mentioned, and depress the upper part of the body. If by coma or asphyxia, use artificial respiration, produced by the hands or electricity.

The use of anæsthesia, in the practice of medicine, was then shortly reviewed, and shown to be chiefly attended with benefit in relieving pain, however arising. Its employment in many diseases, implying lesions of sensation and motion, was dwelt on, together with its use in cases of mental affections. In the paroxysms of many spasmodic and neuralgic affections, it was shown to be invaluable. Surgery, however, was declared to be the real province of anæsthesia, and that in which its benefits were more gratefully recognized. The advantages accruing to both the surgeon and the patient were pointed out, and the cases in which it was employed were stated to be reducible to those in which pain or spasm were to be allayed. Dr. M'Leod emphatically denies that there was anything in gun-shot wounds which made the use of anæsthesia in these less beneficial than in the same accidents of civil life, and he contended that as the pain and suffering in these cases were very great, so much the more necessity existed for its use. He stated his conviction that the mental state of the patients, who were the recipients of these two species of injuries, made no real difference in this question. Shock and pain were both the most fruitful causes of a fatal issue both in primary and secondary operations; and as these two evils were avoided by the employment of anæsthesia, we should naturally expect to find that the mortality succeeding capital operations had decreased since the use of anæsthesia had become general, and this the author would presently show was the fact. In the examination, adjustment, and dressing of injuries; in the employment of instruments to cure disease; in the reduction of hernia and dislocations; and, in short, in all those instances in which the surgeon's interference caused pain, or in which it was desirable to prevent any muscular opposition on the part of the patient, the use of anææsthesia was shown to be invaluable. Its use in tetanus during the war was spoken of, and the fact stated that in one well-marked case at least its continued used had been followed by recovery. Dr. M'Leod thought that in the General Hospital its use had been, beyond all question, successful, and he did not agree with Mr. Mouat, who had read a paper on the subject at a former meeting, that in the cases there or elsewhere it could be fairly said to have produced any disagreeable consequences. The very few cases in which it had been said to have given rise to unpleasant or fatal effects contrasted strongly with the multitude in which it had been successful, in which it had obviated pain and saved life. The writer next glanced at the various objections which had at various times been made to the use of anæsthesia, and showed how false both theory and practice had proved them to be. He also alluded to the many operations which were now practicable and hopeful, which, before the discovery of anæsthesia, were unattainable. To military surgeons, the detection of feigned disease was now a matter of simplicity, and many of the questions which divided them in opinion were now much changed in their bearings. All objections to primary amputations were now set aside, and the doctrine of 'making the knife follow the ball,' had received a new and important support. The writer expressed his strong conviction that shock was not established till some time (the direction being different in different injuries and persons) after the receipt of an injury, as by a ball, and he said he felt sanguine that an operation under chloroform, performed in this interval, would obviate much of the succeeding shock, by removing its cause. He was sorry that during the past seige, which was so manifestly favorable for testing this, so few attempts had been made to carry it out. In conclusion, the author having stated his opinion that no case absolutely forbade the use of chloroform, referred to those in which its administration should be carefully watched. Operations on the back of the mouth, from the danger of blood getting into the throat; cases of severe hemorrhage, or long suppuration from the activity of the absorption; acute disease of the lungs from the irritation caused; disease of the heart, particularly in active dilitation, with weakening of the organ, on account of the fear of fatal syncope; aneurismal disease of the aorta, or marked apoplectic diathesis; and cases in which fatty degeneration may be suspected. These seem to comprise all those cases in which extra caution was necessary. That care should be taken that the agent employed should be pure was insisted upon, and the tests to determine the presence of adulterations were stated. Dr. M'Leod next gave the statistics furnished by Mr. Skey, Drs. Simpson and Snow, and MM. Velpeau and Bouissau, as affording a large amount of evidence in favor of chloroform in surgery, as not only proving its beneficial influence in relieving pain, but in directly saving human life; and he stated that while, during the past war, it had been administered in innumerable instances, only one death had followed its use—and in that case the patient was not placed in the recumbent posture. While expressing his own belief, that, if administered with proper caution, chloroform might with perfect safety be employed in all those cases which fall to the care of the military surgeon, in which it is desirable to overcome pain or spasm, or muscular exertion, the author called on the members of the Society to give a clear and decided verdict on this important subject, founded on the experience of this great war, which would forever put this question at rest, and remove all doubts as to their appreciation of the immense benefits bestowed on humanity by anæsthetics.

Dr. Blenkins understood the author to say, that only one fatal case had taken place, whereas he believed more had occurred. He felt very much obliged to Dr. M'Leod for a highly interesting paper, and considered the author had gone most fully into his subject. He (Dr. Blenkins) trusted that each individual would give his experience of the use of chloroform, without any view to opposition, but for the benefit of all. In his practice, chloroform had been just as successful in the Crimea, in severe operation, as at home; and he quite agreed with the author, that there is no difference in the effects of accidents in civil and military life—he had not seen any ill effects in any one of his cases. He did not regard chloroform as a drug to be treated with carelessness and indifference, but with great care; we should watch the pulse and respiration. Objections should not be made to its use in easy operations without a fatal termination. He remembered one case, that of an old soldier, where the patient was a very long time before he perfectly recovered; but this was owing to the length of time it took to get him under the influence of the drug; and this, again, was accounted for by his addiction to strong drinks. The theory of its action would occupy too much time for him to enter upon now. He expressed his conviction that chloroform acts through the blood, and looked on it as a remedy requiring vigilance in its use. He had operated fifteen times under its influence, besides having given it in tetanus, fits, &c. He believed it to act as a stimulus, and to raise the pulse when low. On one occasion, he was obliged to remove the head of the femur at night, with only one assistant, and he believed it almost impossible to have done so without the aid of chloroform. In conclusion, he begged to state that all his observations agreed with those of the author.

Dr. Sall considered that the Society was much indebted to Dr. M'Leod for the very valuable and lucid paper which he had just read, and as we are not confined, to-day, to the discussion of chloroform in cases of gun-shot wounds, he begged to state that he had used it with the most marked success in cases of delirium tremens, and he had found it a more beneficial mode of treatment than the stimulo-narcotic plan. He then alluded to the case of a youth in a band of the 93d Regiment, who, after suffering much distress of mind from disappointment, relapsed into a state resembling hysteria, accompanied with a complete cataleptic condition, in which he remained for twelve hours. In this case a variety of stimulating plans of treatment was tried without success, but under the anæsthetic use of chloroform the boy quite recovered. He considered that in the administration of chloroform there were two things necessary to be borne in mind—the purity of the drug, and the correct mode of administering it. He (Dr. Sall) had never witnessed an unfortunate result from its use.

Dr. Bowen had never heard a more practical paper, and he agreed entirely in the views of the author. He had seen anæsthetic agents used for the last six years, and had given them himself between two thousand and three thousand times. He considered that the best mode of administration, was by means of a napkin, as now stated by Dr. M'Leod. As regarded the purity of the drug, he was only surprised that more accidents had not happened from its frequent impurity. He had detected both free chlorine and pure muriatic acid in different samples furnished twelve months since to the Military Hospital at Plymouth. It had been given by himself for fourteen hours to one woman; and Professor Simpson, in a case of convulsions in an infant, had given one hundred ounces within a period of two weeks, with most beneficial results. He had been informed by the Russians, that throughout the seige chloroform had been used with only one or two fatal results, which was not surprising under the circumstances: it appeared that their mode of administering it was the same as that now recommended.

Mr. Howard considered that in delirium tremens it was invaluable; by its means he had procured sleep, when opium could no longer be given with safety, and after every other means had absolutely failed.—*Lancet.*

